# Draft Genome Sequence of Aspergillus lacticoffeatus WU-2020, a Citric Acid Producer Suitable for Solid Culture That Belongs To Aspergillus Section *Nigri*

**DOI:** 10.1128/mra.01093-22

**Published:** 2023-01-04

**Authors:** Isato Yoshioka, Hiroki Takahashi, Yoko Kusuya, Takashi Yaguchi, Akira Shibata, Kohtaro Kirimura

**Affiliations:** a Research Institute for Science and Engineering, Waseda University, Tokyo, Japan; b Medical Mycology Research Center, Chiba University, Chiba, Japan; c Molecular Chirality Research Center, Chiba University, Chiba, Japan; d Plant Molecular Science Center, Chiba University, Chiba, Japan; e Faculty of Science and Engineering, Waseda University, Tokyo, Japan; Vanderbilt University

## Abstract

Aspergillus lacticoffeatus WU-2020 is a citric acid hyperproducer that is suitable for solid culture. Here, we present a high-quality draft of its genome sequence (35.9 Mb), which consists of 11 scaffolds and contains 11,490 genes. We also present the mitochondrial genome, which is 31.3 kb in length.

## ANNOUNCEMENT

**FIG 1:**
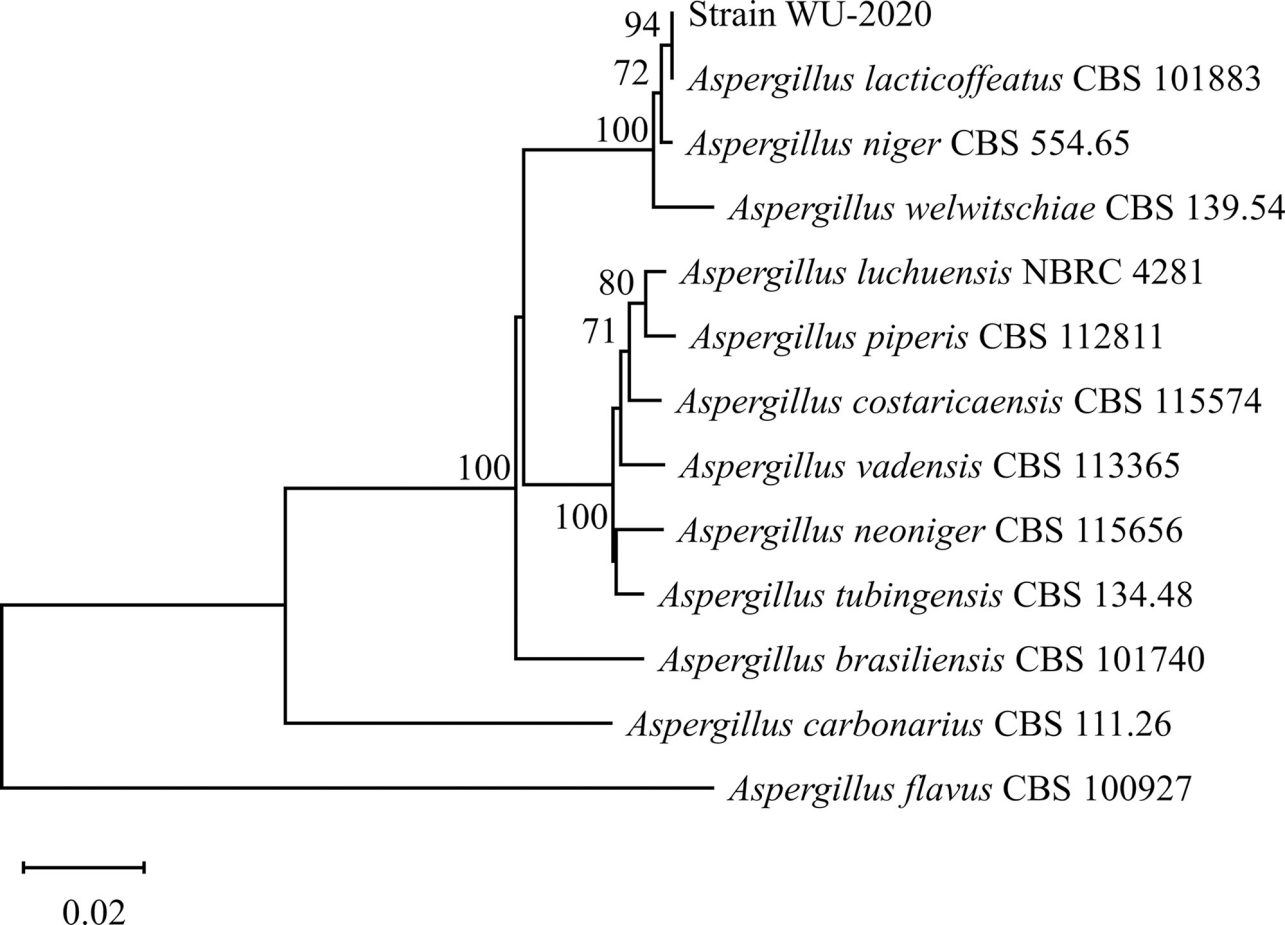
Phylogenetic tree of strains belonging to Aspergillus section *Nigri*, including strain WU-2020. The tree was generated with the neighbor-joining method by MEGA11 (28) using the combined sequences of rDNA ITS, β-tubulin, and calmodulin; bootstrap values (*n* = 1,000) above 60 are represented on the branches. Scale bar refers to a phylogenetic distance of 0.02 nucleotide substitutions per site. The nucleotide sequences and calculation method reported in previous studies were applied with some modifications (5, 6). Aspergillus flavus CBS 100927 was used as an outgroup strain. Additionally, Aspergillus carbonarius CBS 111.26 was used as a reference strain in the Aspergillus section *Nigri* but not in the A. niger clade.

Citric acid (CA) is a versatile organic acid that is produced via industrial fermentation by filamentous fungal strains belonging to Aspergillus section *Nigri* (1). We previously reported the genome sequence of Aspergillus tubingensis WU-2223L (2), a CA hyperproducer in submerged culture and a mycotoxin nonproducer (3). Recently, we isolated a CA-hyperproducing fungal strain for solid culture from soil in Japan. In brief, a loopful of the soil samples collected was suspended in 5 mL saline (pH 6.5), and 0.1 mL of the suspension was spread on a Czapek-Dox agar plate containing 30 g/L glucose as the sole carbon source. CA-producing fungal strains were selected using agar plates containing bromocresol green as an indicator to reflect acidogenesis, according to a previously described method (4). Among the isolated strains, WU-2020 was selected because it showed the greatest amount of CA production. Strain WU-2020 was maintained on potato dextrose agar at 25°C for 7 days, and the morphology of the colonies was monitored. The genomic DNA was extracted with the ISOPLANT kit (Nippon Gene, Toyama, Japan), according to the manufacturer’s instructions, and partial DNA sequences were determined. Strain WU-2020 was identified as Aspergillus lacticoffeatus, a species of Aspergillus section *Nigri*, based on multigene sequences, i.e., rDNA internal transcribed sequence (ITS), calmodulin, and β-tubulin, by following the method reported previously (5, 6), as shown in Fig. 1. Strain WU-2020 produced 2.1 times more CA than did the type strain CBS 101883 during 4 days on a typical medium (SS medium) for semisolid CA production with bagasse as the carrier (7), as shown in Table 1. A. lacticoffeatus WU-2020 was deposited in the National Institute of Technology and Evaluation Biological Resource Center (as NBRC115414) and the Medical Mycology Research Center, Chiba University (through the National Bioresource Project) (as IFM 65579). Here, we present its draft genome sequence with the aim of understanding the mechanisms underlying CA hyperproduction. Strain WU-2020 was cultured in potato dextrose broth at 25°C for 1 day. The genomic DNA was extracted from the mycelia using phenol-chloroform extraction and a Genomic-tip 20/G kit (Qiagen, Hilden, Germany). DNA libraries for Illumina and Oxford Nanopore Technologies (ONT) sequencing were prepared using a NEBNext Ultra II FS DNA library preparation kit for Illumina (New England Biolabs, Ipswich, MA) and a short-read eliminator kit (Nippon Genetics, Tokyo, Japan), respectively. Paired-end (PE) sequencing (2 × 150 bp) on the HiSeq X platform (Illumina, San Diego, CA, USA) was performed by BGI (Kobe, Japan). The raw PE reads were filtered by eliminating reads with Q scores of <20 or lengths of <40 bases using fastp v0.20.1 (8), which yielded 20,713,137 PE reads (genome coverage, 169×). The ONT long reads were base called with ONT Guppy v4.0.14 and filtered with Porechop v0.2.4 (https://github.com/rrwick/Porechop) and NanoFilt v2.7.1 (9).

**TABLE 1:** CA production in semisolid culture by Aspergillus lacticoffeatus strains

Strain	Glucose consumption (g/L)^*a*^	CA production (g/L)	Yield (wt/wt) (%)^*b*^
CBS 101883	94.6	40.4	43
WU-2020	120	85.2	71

a Cultivation conditions for semisolid culture are shown in reference 7. The initial concentration of glucose is 140 g/L, and bagasse is used as the carrier for cultivation medium for semisolid culture (7).

b The yield is the ratio of the amount of CA produced to that of glucose consumed.

Hybrid *de novo* genome assembly, using short and long reads, was performed according to the method reported by Saud et al. (10), with some modifications. Briefly, 95,487 ONT reads (2.05 Gb) with a mean length of 21,419 bp, corrected using FMLRC (11) and trimmed using Canu v1.9 (12), were assembled using Flye v2.7 (13). The generated contigs were polished using Minimap2 v2.17 (14), Racon v1.4.13 (15), and Medaka v0.11.5 (https://github.com/nanoporetech/medaka), followed by iterative polishing using BWA-MEM2 v2.0 (16) and Pilon v1.23 (17). The gene annotation was performed with Funannotate v1.7.4 (https://github.com/nextgenusfs/funannotate). Funannotate *ab initio* prediction was performed with the option –busco_seed_species=aspergillus_oryzae by AUGUSTUS v3.3.2 (18), GeneMark-ES v4.38 (19), GlimmerHMM v3.0.2 (20), and SNAP v2006-07-28 (21) using exon hints from the proteins of Aspergillus niger (GenBank accession number PRJNA19275) and Aspergillus oryzae (GenBank accession number PRJNA28175) and the UniProt database. RNAs were predicted using tRNAScan-SE v2.0.5 (22) and Barrnap v0.9 (https://github.com/tseemann/barrnap). The mitochondrial genome was analyzed using NOVOPlasty v4.2 (23) and GeSeq v2.03 (24).

The draft genome of A. lacticoffeatus WU-2020, which consists of 11 scaffolds with a total length of 35.9 Mb (G+C content, 49.51%), contains 11,490 protein-coding genes, 273 tRNAs, and 80 rRNAs. The BUSCO score (25) based on the Eurotiales database was 98.6%. The mitochondrial genome (31,324 bp) contains 15 genes, 26 tRNAs, and 2 rRNAs. Genome sequencing revealed that strain WU-2020 possesses putative biosynthetic gene clusters for the mycotoxins ochratoxin A and fumonisin B_2_ (2, 26), similar to the type strain A. lacticoffeatus CBS 101883 (27). Therefore, further studies are required to assess the biosafety of WU-2020 for industrial use.

### Data availability.

The whole-genome and mitochondrial genome sequences of A. lacticoffeatus WU-2020 were deposited in DDBJ/EMBL/GenBank under accession numbers BQMC01000001 to BQMC01000011 and LC670769, respectively. The raw sequencing reads were submitted to the DDBJ Sequence Read Archive under accession number DRA013207.
